# Low-grade fibromyxoid sarcoma around the knee involving the proximal end of the tibia and patella: A rare case report

**DOI:** 10.3892/ol.2014.1888

**Published:** 2014-02-17

**Authors:** JEETENDRA BAJPAI, SAURAV SHUKLA, MOAZZAM JAH, ALOK KUMAR SINGH, MOHIT GOEL, AMIT MOURYA, NIKHIL SACHDEVA

**Affiliations:** Department of Orthopaedics, Vivekanand Polyclinic and Institute of Medical Sciences, Lucknow, Uttar Pradesh 226016, India

**Keywords:** low-grade fibromyxoid sarcoma, tibia, patella

## Abstract

Low-grade fibromyxoid sarcoma (LGFMS) is a distinctive variant of fibrosarcoma. LGFMS is a rare soft tissue tumor that tends to develop in the deep soft tissue of young adults and has the potential for local recurrence or distant metastasis. The current case report presents a 22-year-old male complaining of a slow growing painless mass in the right knee over a period of 10 years. Following complete evaluation by radiological and histopathological examination, a diagnosis of LGFMS was confirmed and a wide excision was performed. Currently, the patient has been under follow-up for the last five years without any evidence of metastasis. The present case report provides further information concerning the diagnosis, imaging and management of LGFMS.

## Introduction

Low-grade fibromyxoid sarcoma (LGFMS) is a distinctive variant of fibrosarcoma (1). LGFMS is a rare soft tissue tumor that tends to develop in the deep soft tissue of young adults and has the potential for local recurrence or distant metastasis (2). The majority of LGFMSs occur in a subfascial location, however, on rare occasions the subcutis or dermis may be affected (3). These fibroblastic tumors typically occur in the lower limb/groin area, however, sporadically occur in other deep soft tissues. In a previous study by Evans (4), there was an even distribution of male and female patients, yet a marked male preponderance was observed. The ages ranged between six and 51 years, although the majority were young adults (ages, 25–46 years). The majority of the LGFMSs were well-circumscribed but not encapsulated, therefore, the resections were often incomplete (5). This study was approved by the ethics committee of Vivekanand Polyclinic and Institute of Medical Sciences (Lucknow, India).

## Case report

**Figure 1:**
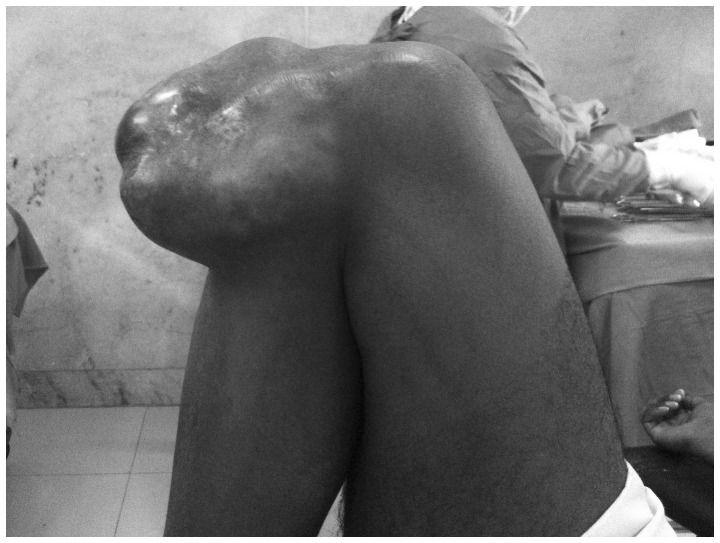
Image showing full flexion of the knee.

**Figure 2:**
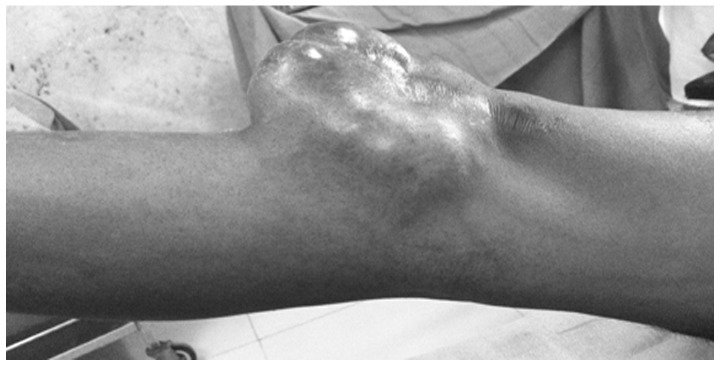
Image showing full extension of the knee.

A 22-year-old male was admitted to the Department of Orthopaedics (Vivekanand Polyclinic and Institute of Medical Sciences, Lucknow, India) complaining of a slow growing painless mass in the right knee that had developed over a period of 10 years. Physical examination demonstrated a mass on the anterior medial aspect of the right knee, which was not tender or mobile, however was rubbery and hard in consistency. Full flexion and extension was observed without any restriction of joint movement (Figs. 1 and 2).

**Figure 3:**
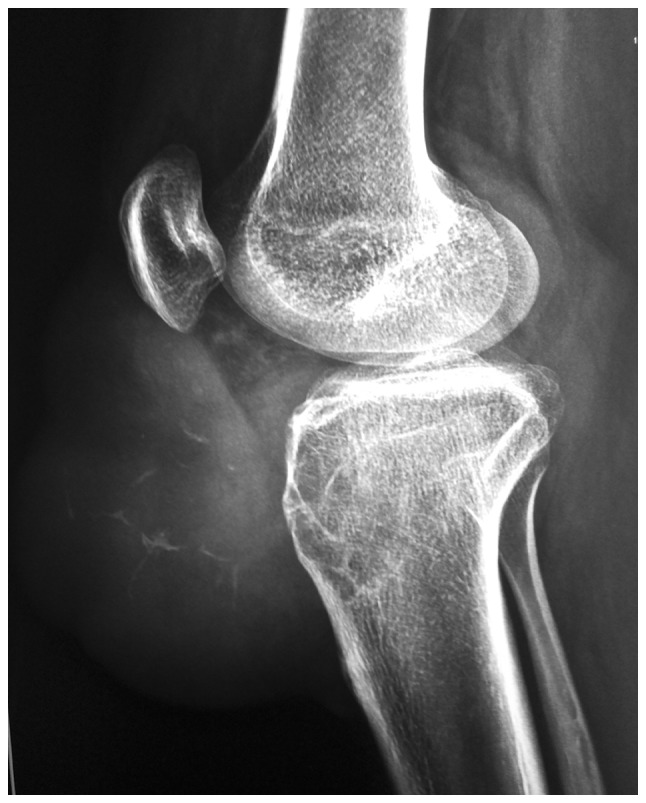
Lateral view X-ray showing a lytic area in the proximal end of the tibia presenting with soft tissue calcification.

**Figure 4:**
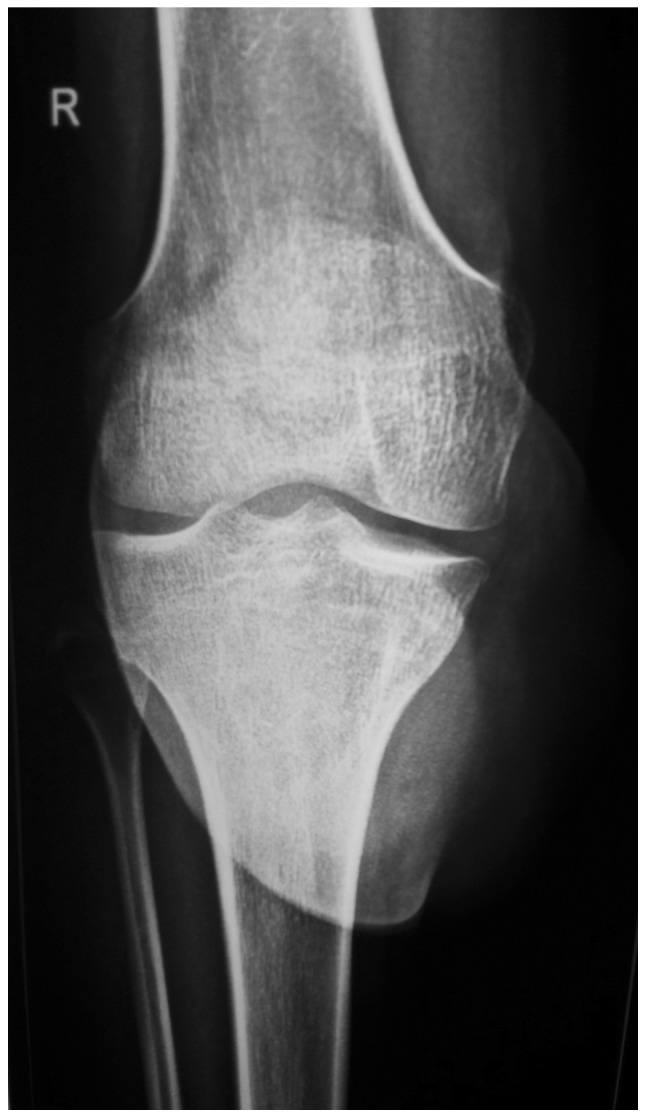
Anteroposterior view X-ray showing a normal joint space and a soft tissue shadow.

The laboratory investigations were unremarkable. A plain radiograph showed a lytic lesion in the anteromedial proximal tibia and a large soft tissue shadow presenting with slight calcification (Figs. 3 and 4).

**Figure 5:**
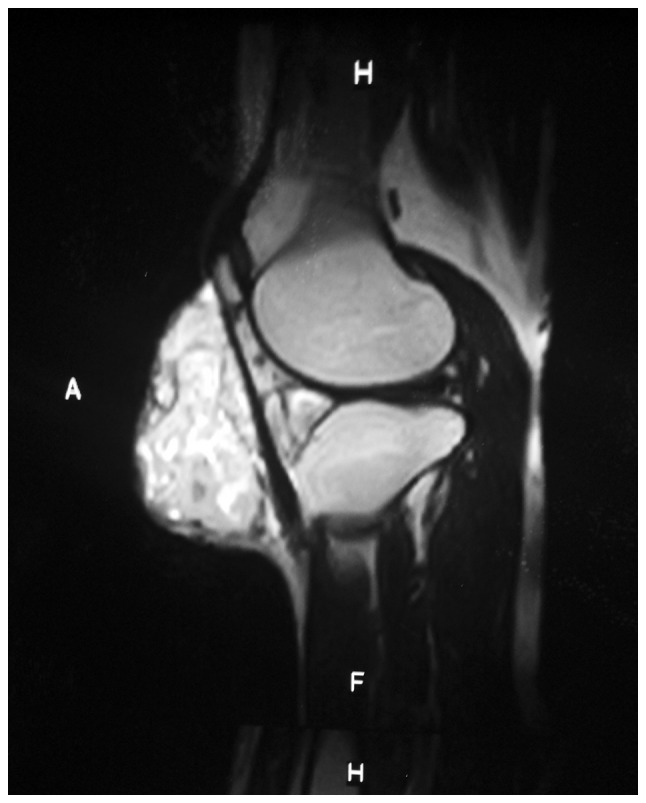
T1 fat-suppressed contrast-enhanced magnetic resonance imaging revealed a hyperintense central section and hypointense irregular wall, corresponding to a myxoid and fibrous pattern.

**Figure 6:**
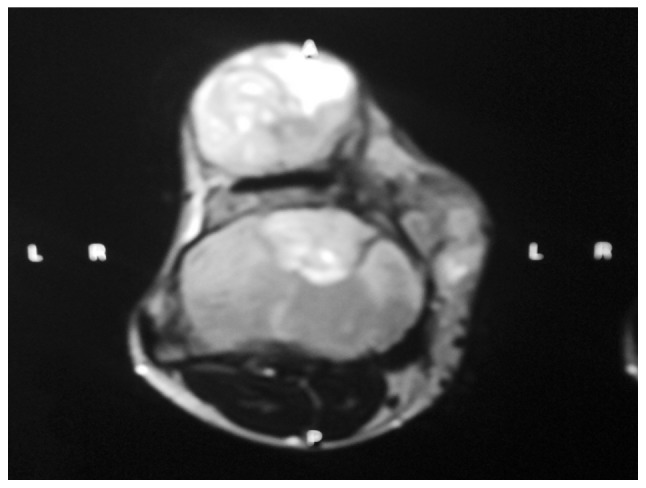
Magnetic resonance image showing the axial section incorporating the anteromedial portion of the tibia.

Magnetic resonance imaging (MRI) revealed that the tumor was 14×12×9 cm in size, incorporated the periosteum and anteromedial aspect of the tibia, and was beneath the synovium and periosteum of the anterior margin of the patella. The MRI evaluation of the area also demonstrated a mixed myxoid and fibrous pattern within the tumor. The tumor matrix was partially calcified and relatively well-defined with an irregular low signal intensity on the T1-weighted image and heterogeneous low and high signals on the T2-weighted image (Figs. 5 and 6).

Subsequently, the patient underwent a wide biopsy to acquire additional information prior to surgical resection of the mass. Histopathological and immunohistochemical evaluations were performed to examine the neoplastic cells and a diagnosis was confirmed. As a result of the clinical and radiographic observations, malignancy was not ruled out and a low-grade mesenchymal neoplasm was considered.

A wide excision of the tumor was performed four weeks later. The mass was well-circumscribed, however, it was not encapsulated, thus, there was uncertainty with regard to the surgical margins of the resection. Thus, 5-cm excisions of the proximal tibia from the tumor margins, patella, synovium, muscles and patellar tendon surrounding the tumor mass were obtained, and sent for histopathological examination.

**Figure 7:**
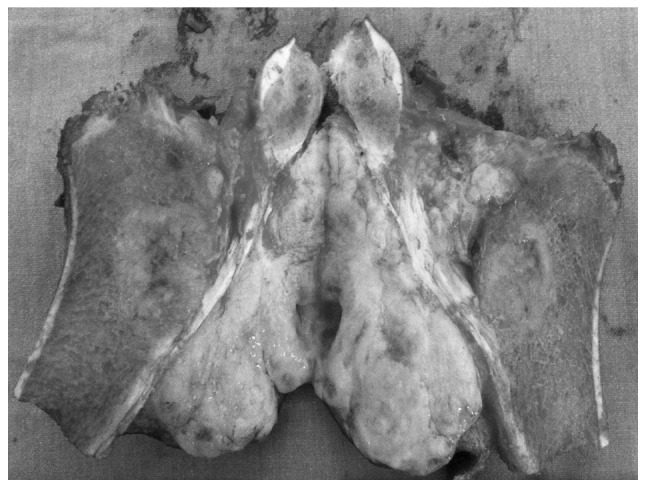
Cut section of the gross specimen.

The cut (excised) section of the gross tumor showed a well-circumscribed, lobulated, round and firm mass. The excised surface tissue was yellow-white in color with a focal gelatinous appearance and an elastic consistency; furthermore there was a viscous seromucinous fluid. In addition, the excised surface was fibrous to myxoid without any areas of hemorrhaging or necrosis. A gritty sensation was experienced while cutting the tumor, which invoved the periosteum, the antero-medial aspect of proximal tibia and the inferior pole of patella (Fig. 7).

**Figure 8:**
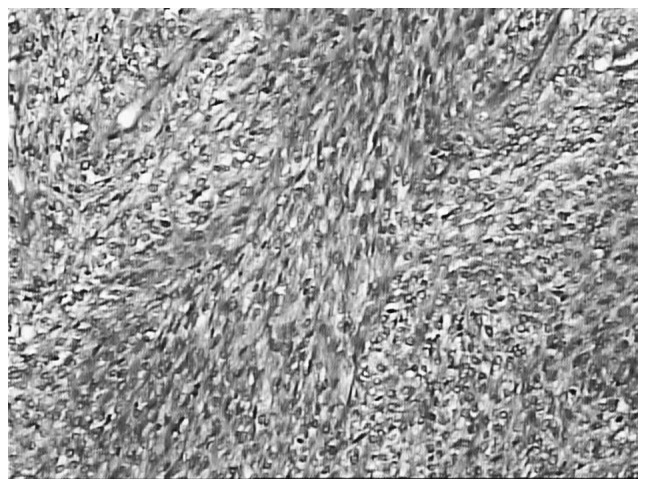
Microscopic image. The tumor was composed of bland spindle shaped cells with small hyperchromatic nuclei which were oval to tapered in shape, containing finely clumped chromatin with palely eosinophilic cytoplasms. The cells exhibited a mild nuclear pleomorphism with little mitotic activity. Hematoxylin and eoisin staining. Magnification, ×200.

Microscopic examination of the tumor identified alternating fibrous and myxoid components; the myxoid area demonstrated tumor cells in a whirling growth pattern with capillary-sized blood vessels (Fig. 8).

The proximal fibula (~6 cm) was removed as docking was to be performed on the distal femur, for which a notch was created in the intercondylar area to accommodate the tibia. The distal femur and tibia were stabilized using two Kirschner-wires, and bone grafting was performed using the femoral condyles following the removal of the articular cartilage.

The limb was stabilized via Ilizarov fixation followed by corticotomy of the distal tibia with distal fibulectomy, as shortening of >6 cm was identified following arthrodesis of the knee joint and limb length preservation was a predominant concern for the patient. No perforation of the vessels or nerves was evident during the mass removal. Following surgery, the patient experienced no major complications and the neurovascular status of the patient remained intact. At present, the patient has been regularly followed up for five years; every three months for the first year, six monthly for the following two years and annually for the last two years. No clinical or radiological evidence has been identified indicating recurrence or metastasis.

## Discussion

LGFMS is a distinctive variant of fibrosarcoma with a high metastasizing potential and, occasionally, long intervals between tumor presentation and metastasis are observed (1). LGFMS is recognized as an uncommon soft tissue neoplasm (6). These tumors have previously been described in numerous locations, including the chest wall, axilla, shoulder, inguinal region, buttocks, neck and thigh (7). Although a large LGFMS has been reported to have arisen from the abdominal wall of the falciform ligament (8), LGFMS has not previously been reported around the knee, and involved the proximal end of the tibia, synovium, periosteum of the patella and patellar tendon.

Originally, LGFMS was reported by Evans in 1987 (1), who subsequently described 10 additional cases in 1993 (4). Thus far, a few sporadic case reports and series have been reported (9). The predominant pathologies to be considered in a differential diagnosis include desmoid fibromatosis, peripheral nerve sheath tumor myxoid liposarcoma, spindle cell liposarcoma and low-grade myxofibrosarcoma (MFS) (5).

Macroscopically, LGFMS is a well-circumscribed and encapsulated mass, indicative of a benign lesion. The excised surfaces of such tumors exhibit a firm, fibrous, yellow-white appearance with glistening areas secondary to the accumulation of a myxoid ground substance (1).

The microscopic appearance of LGFMS is somewhat variable, as the name indicates, consisting of bland fibroblasts with a whorled or linear arrangement, alternating between less cellular areas and myxoid stroma. The tumor cells tend to be small, with poorly defined, pale eosinophilic cytoplasms and round or ovoid nuclei. Nucleoli are absent or indistinct and the mitotic figures tend to be absent or sparse. In addition, nuclear anaplasia and necrosis are generally absent, although, one case in a study by Evans were identified as ‘dedifferentiated’, in a 30-year follow-up, with sheets of anaplastic rounded cells (4).

Immunohistochemical staining for LGFMS is only positive for vimentin and is negative for a variety of antibodies, including desmin, keratin, S100 protein, epithelial membrane antigen, CD34 and CD31. Muscle-specific actin is positive in the wall of small vessels within the tumor and markedly positive in the peripheral fibrous layer (11). Ten years after the first description of LGFMS by Evans (1), a similar pathological entity, hyalinizing fibrosarcoma with giant rosettes (HSTGR), was identified. In addition, a following cytogenetic report demonstrated an identical recurrent t(7;16)(q34;p11) translocation and fusion between the FUS and CREB3L2 genes in LGFMS and HSTGR, which was confirmed by multiple studies regarding these subtypes of fibrosarcoma, thereby providing genetic proof that these two tumors are variants of the same entity (12). Furthermore, the FUS/CREB3L1 fusion transcripts of LGFMS may be reliably detected in paraffin-embedded tissues using reverse transcription-polymerase chain reaction (13).

LGMFS must be distinguished from MFS, the most common sarcoma affecting the limbs of elderly patients and its high-grade end of the spectrum is considered to be a myxoid variant of malignant fibrous histiocytomas (MFH) by previous authors (14,15). MFS comprises of a wide morphological spectrum; high-grade lesions tend to form solid sections with a continuous transition to a storiform-pleomorphic type MFH. Continuity between high- and low-grade areas of MFS has previously been indicated by the presence of solid high-grade components within low-grade tumors, as well as the progression of a subset of low-grade MFS into high-grade tumors in local recurrences (16). Owing to its ultrastructural features, which closely resemble ordinary fibroblasts, previous studies have favored the nomenclature of MFS instead of myxoid MFH. MFS was regarded as a distinct fibroblastic neoplasm that is characterized by a myxoid nodular appearance, and curvilinear vasculatures with a considerably broad spectrum of nuclear pleomorphism, cellularity and mitoses (17).

Diagnosis of LGFMS or HSTGR is not difficult if the tumor is removed completely, and sent for histopathological and immunohistochemical staining, demonstrating characteristic morphological and immunophenotypic features as previously described. For an exact diagnosis, it is recommended that the patient receives an open or excisional biopsy as occasionally, material obtained from a fine-needle aspiration or core biopsy is not sufficient and leads to misdiagnosis. If a myxoid pattern is present, the patient must be referred to a cytogenetics department to exclude rare LGFMS (3).

Although imaging observations of LGFMS are non-specific, certain computed tomography (CT) and MRI observations have previously been described. On non-contrast CT images, the fibrous component of these tumors has been described as isodense or muscle tissue and the myxoid component has been described as hypodense (18). Several previous case reports described the variable MRI observations in patients with LGFMS (19). In general, fibrous tissue components appear hypointense on T1- and T2-weighted images and show marginal enhancement. Myxoid tissue components are hypointense on T1-weighted images, hyperintense on T2-weighted images and show variable enhancement on contrast-enhanced T1-weighted images (18). Occasionally, calcification may also be identified within the tumors (11). Fibromatosis is usually hypointense on T2-weighted images, however, may also show heterogeneous hyperintensity, reflecting marked cellularity or myxoid tissue. In addition, neurogenic tumors are characterized by the entry and exit of nerves (19).

The clinical presentation of LGFMS is usually long-standing and is predominantly associated with the anatomic location of the mass. LGFMS usually presents as a painless soft tissue mass with a prebiopsy duration of more than five years in 15% of patients that are diagnosed with benign lesions and local recurrence in 68%, metastasis in 41% and mortality in 18% of the patients (20). In an additional study, the patients exhibited a recurrence rate of 54%, metastasis rate of 6% and mortality rate of 2%. In the same study, the presence of focal areas of high cellularity, nuclear enlargement, increased mitotic activity and necrosis were found to be of no prognostic significance for recurrence or metastasis (21).

LGFMS has previously been reported to recur and metastasize, and has been found to recur as early as six months to as late as 50 years in 65% of patients. Metastasis has also been reported to occur frequently, with the lungs as a common site. These tumors have a protracted course even subsequent to metastasis (8).

Once the diagnosis of LGFMS or HSTGR has been determined, a full oncological assessment is required. This must include a CT scan of the chest as metastasis to the lung is the most common scenario. Due to the high risk of late metastasis, a clinical follow-up and chest imaging must be performed for an extended period of time. However, how regularly imaging of the chest must be repeated remains unclear (3). The current study presented a case of rare LGFMS around the knee, involving the proximal end of the tibia, synovium, periosteum of the inferior pole of the patella and the patellar tendon. The patient was treated with wide excision (5 cm) of the proximal tibia and fibula from the tumor margin, along with the patella and synovium. This was followed by docking of the tibia, for which a notch was created in the intercondylar area of the distal femur. In addition, Ilizarov fixation was used to stabilize the limb. Fibulectomy and corticotomy was performed to maintain limb length.

In conclusion, little is known concerning LGFMS, its occurrence in and around the joints, the involvement of the bone and its definitive treatment. However, the current case report presents further information regarding the diagnosis, imaging and management of LGFMS. There is no dedicated protocol for the early diagnosis, treatment and follow-up of LGFMS, however, it is associated with a high incidence of recurrence and metastasis after a long duration. Following a long latent period, all LGFMS must be treated as a malignant tumor and, thus, undergo a wide excision, followed by a full oncological assessment and follow-up. This must include a CT scan of the chest, as metastasis to the lung is the most common scenario. Additionally, due to a high risk of metastasis, a clinical follow-up and chest CT scan must be performed over an extended period, as it remains unclear how frequently chest CT scans must be repeated.
